# Verification measurements of an eMC algorithm using a 2D ion chamber array

**DOI:** 10.1120/jacmp.v17i5.6150

**Published:** 2016-09-08

**Authors:** Mark D. Wanklyn, Ghirmay Kidane, Liz Crees

**Affiliations:** ^1^ Medical Physics Department Guy's & St. Thomas' NHS Foundation Trust London UK; ^2^ Radiotherapy Physics, Barking Havering & Redbridge University Hospitals NHS Trust Romford Essex UK

**Keywords:** electron, Monte Carlo, eMC, verification, ion chamber array

## Abstract

The aim of this study was to assess the suitability of the Im'*RT* MatriXX 2D ion chamber array for performing verification measurements on the Varian Eclipse electron Monte Carlo (eMC) algorithm for a range of clinical energies (6, 12, and 20 MeV) on a Varian 2100iX linear accelerator. Firstly, the suitability of the MatriXX for measuring percentage depth doses (PDD) in water was assessed, including characterization of the inherent buildup found in the MatriXX. Secondly the suitability of the MatriXX for measuring dose distributions in homogeneous and heterogeneous phantoms was assessed using gamma analysis at 3%/3 mm. It was found that after adjusting the PDD curves for the inherent buildup, that the position of $R_{50,D}$ measured using the MatriXX agreed to within 1 mm to the PDDs generated using the eMC algorithm for all energies used in this study. Gamma analysis at 3%/3 mm showed very good agreement (>95%) for all cases in both homogeneous and heterogeneous phantoms. It was concluded that the Im'*RT* MatriXX is a suitable device for performing eMC verification and could potentially be used for routine energy checks of electron beams.

PACS number(s): 87.55.km, 87.55.Qr

## I. INTRODUCTION

The electron Monte Carlo (eMC) algorithm in Eclipse v.11 (Varian Medical Systems, Palo Alto, CA) is a fast implementation of a Monte Carlo simulation of the dose deposition of high‐energy electron beams. The eMC algorithm consists of two models; the Initial Phase Space (IPS) model,^(1)^ which models the electron beam emerging from the treatment head, and the Macro Monte Carlo (MMC) Transport model,^(2)^ which calculates the dose deposition in the material as the electron travels through the material. The eMC algorithm models each individual primary electron interaction along with secondary electron interactions, and for that reason can require both a large amount of computer processing power and long calculation times in order to achieve an accurate dose distribution.^(3)^

The accuracy of the Eclipse eMC algorithm has been evaluated in terms of optimal calculation parameters,^(4)^ accuracy in heterogeneous and homogeneous phantoms,^(5)^ radiochromic film verification,^(6)^ and small field dose calculation.^(7)^ Currently, radiochromic film is used to perform eMC verification measurements and has been shown to agree within 2%/1 mm with measurements taken using an ionization chamber, and 2%/2 mm with an eMC algorithm.^(8)^ However, using radiochromic film can require significant time to prepare the film for use and to read out the resulting exposure.^9^, ^10^ There is also the requirement to calibrate each batch of film and the scanner, as well as to develop a suitable scanning protocol. Another problem with radiochromic film is that each sheet is single‐use and therefore high costs can be incurred when using radiochromic film to perform eMC verification measurements. Unlike radiochromic film, 2D ion chambers are multi‐use, provide an instant readout which can be analyzed on set, and have a constant calibration factor that can be stored, removing the need for repeat calibrations. Most radiotherapy departments will already have a 2D ion chamber array available which is typically used to perform QA measurements, therefore there is no further cost incurred in performing eMC verification measurements. This paper assesses the suitability of using a 2D ion chamber array to perform eMC verification measurements in homogeneous and heterogeneous solid water phantoms.

## II. MATERIALS AND METHODS

### A. The Varian Eclipse eMC algorithm

In order to configure the IPS and transport models in the treatment planning system (TPS), measured beam data are required. This data include open‐field (no electron applicator in place) and applicator measurements. The data required for the configuration of the eMC algorithm are outlined in detail in the Varian Eclipse algorithm guide and in the literature.^(11)^ The measurements required by the Eclipse treatment planning system include PDD curves in water for 6, 9, 12, 16, and 20 MeV with all available applicator sizes of 6×6,6×10,10×10,15×15, and $20\times 20\;{\text{cm}}^{2}$. Additionally, point‐dose measurements in water for all energy/applicator combinations listed and open‐field (no applicator in place) measurements are required for the following: PDDs in water for all energies; in air profiles at 95 cm SAD and point‐dose measurements in water for all energies at the reference depth. The configuration process then involves fitting monoenergetic beam data to the measured polyenergetic beam data provided. The eMC algorithm will use the fitted monoenergetic beam data in all calculations.

The eMC algorithm has user‐selectable calculation parameters which determine the overall accuracy of the calculation. These have been explored in the literature^4^, ^11^ and the parameters used in this study are shown in Table 1. These values are based upon the recommendations set in the paper by Zhang et al.^(11)^ and allow for eMC calculations to be performed in a clinically acceptable timeframe whilst maintaining accuracy to ensure a clinically relevant result.

**Table 1:** Calculation parameters used throughout the study for all energies. These parameters allow for accurate dose calculations in a clinically acceptable time.

*Calculation Parameter*	*Value*
Accuracy (%)	1
No. Particle Histories	0
Calculation grid size (cm)	0.2
Smoothing method	3D Gaussian
Smoothing level	Medium
Random generator seed number	Default

### B. The MatriXX 2D ion chamber array:

The MatriXX I'm*RT* (IBA Dosimetry, Schwarzenbruck, Germany) 2D ion chamber array is an array of 1,020 parallel plate chambers (0.08 cm^3^ volume) arranged in a 32×32 grid with a detector center‐to‐center spacing of 7.62 mm, giving a maximum active area of $24.4\times 24.4\;{\text{cm}}^{2}$. The MatriXX has an inherent buildup of 3 mm of Tecaran ABS plastic. The software used in conjunction with the MatriXX is Omnipro Im'*RT* (IBA Dosimetry)

The MatriXX was calibrated for absolute dose measurements for the range of electron energies used in this study (6, 12, 20 MeV). Restrictions in access to the linear accelerators limited the amount of data that could be collected and therefore these energies were chosen to represent the range of energies available in the clinic, whilst minimizing the need for linear accelerator time. The calibration was carried out by comparing the central‐axis reading (interpolated from the readings of the four most central chambers of the MatriXX as it does not have a central axis chamber) on the MatriXX with the absolute dose measured using an NACP chamber. The absolute dose on the NACP chamber was measured for all energies at their respective reference depth ($z_{\text{ref}}$) in a solid water phantom, with3 applicator size $10\times 10\;{\text{cm}}^{2}$ and 100 cm SSD, ($z_{\text{ref}}$ varies with energy, see Table 2). An off‐axis calibration on the Im'*RT* MatriXX was made to compensate for nonuniformity of off‐axis chamber responses.

**Table 2:** $z_{\text{ref}}$ values for each electron energy used in this study.

*Energy (MeV)*	$Z_{\text{ref}}$ (mm)
6	13.6
12	30.1
20	50.0

### C. PDD verification using the MatriXX 2D ion chamber array *C.1 Inherent buildup characterization*

The inherent build‐up of the MatriXX 2D ion chamber array has an equivalent path length in water of 3.6 mm for photons. It has been shown that the path length in water of this inherent buildup varies with electron energy and beam quality.^(12)^ For this study, PDD curves were measured for 6, 12, and 20 MeV, using the MatriXX with different thicknesses of solid water buildup (2 mm step size), and normalized to their maximum values. The position of $R_{100,D},R_{80,D},R_{50,D}$, and $R_{30,D}$, measured using the MatriXX, were compared to those from PDD curves obtained using an NACP chamber in water. Although the detectors in the MatriXX are not NACP chambers they are similar in design, albeit with a smaller active volume. An approximation to NACP chambers was made, and a solid water to water fluence ratio correction of 1.013 was applied to the depths measured in solid water. This fluence ratio correction was measured when absolute output was measured using NACP chambers in solid water. The scaling factor was derived to account for the difference in fluence between water and solid water.

#### C.2 Comparing R50,D measured using the MatriXX 2D ion chamber array vs. calculated using eMC

The calculated shift due to the inherent buildup at each energy was applied to the PDD curve obtained using the MatriXX to allow a direct comparison to the PDD generated using eMC. In order to obtain the PDD using eMC, a plan was calculated on a solid water phantom using the calculation parameters shown in Table 1 and a $10\times 10\;{\text{cm}}^{2}$ applicator for each energy (6, 12, 20 MeV).

The central depth‐dose plane was exported to OmniPro Im'*RT* and the profile along the center of the dose plane plotted.

The depth of $R_{50,D}$ measured using the MatriXX was compared to the $R_{50,D}$ calculated using eMC.

### D. Dose plane verification using the MatriXX 2D ion chamber array

#### D.1 Homogeneous solid water phantom

Dose planes were measured using the MatriXX in a simple homogeneous solid water phantom, with 5 cm solid water backscatter and 2 cm, 5 cm, 7 cm solid water buildup on top of the MatriXX for 6, 12, and 20 MeV, respectively. 100 monitor units (MUs) were delivered using a $10\times 10\;{\text{cm}}^{2}$ applicator at 100 cm SSD.

Equivalent dose planes were planned and calculated using eMC and were then exported to the OmniPro Im'*RT* software.

Using the gamma analysis tool within OmniPro Im'*RT* at 3%/3 mm and a pass rate of 95%γ≤1, the similarity between the eMC calculated and MatriXX measured dose planes was assessed. The ROI where the gamma analysis was performed was the area encompassed by the 20% isodose.

#### D.2 Heterogeneous solid water phantom (with lung insert)

Dose planes were measured using the MatriXX with 5 cm solid water backscatter and a heterogeneous buildup created using the Standard Imaging (Ref 91235) (Standard Imaging Inc., Middleton, WI) lung phantom with an additional 1 cm solid water for all energies in this study. A $10\times 10\;{\text{cm}}^{2}$ applicator at 100 cm SSD was used and 100 MU delivered.

The solid water phantom was CT‐scanned, imported into Eclipse and the corresponding dose planes calculated using eMC. Gamma analysis was performed on the dose planes using the OmniPro Im'*RT* software and the similarity of the dose planes assessed using a criterion of 3%/3 mm and a pass rate of 95% 4gM ≤ 1. The ROI where the gamma analysis was performed was the area encompassed by the 20% isodose.

## III. RESULTS AND DISCUSSION

### A. PDD verification using ion chamber array

#### A.1 Inherent buildup characterization

**Figure 1:**
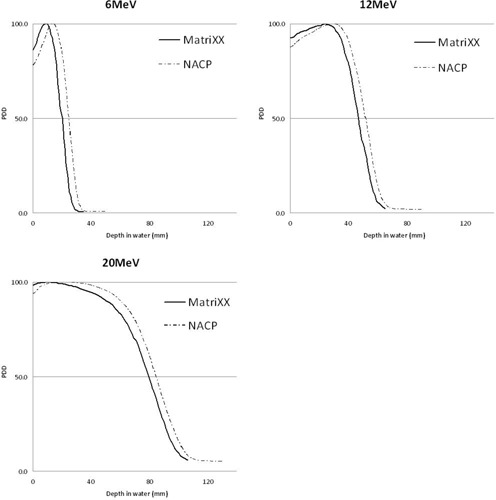
Percentage depth‐dose curves obtained using the MatriXX in solid water (with fluence ratio correction applied) and NACP chamber in water for 6, 12, and 20 MeV showing a shift in PDD curve. The zero position of the MatriXX is not a real zero position but is used to show 0 cm solid water buildup used.

It can be seen from Fig. 1 that there is a shift between the PDD curve measured using the MatriXX compared to the PDD curve measured using the NACP chamber. The average difference in $R_{100,D},R_{80,D},R_{50,D}$, and $R_{30,D}$ position (in mm) between the MatriXX measurements in solid water and the NACP measurements in water was calculated for each energy (6, 12, and 20 MeV) and applied as the inherent buildup to the PDD curve measured using the MatriXX.

This shift was found to be energy dependent at 4.9 mm, 4.9 mm, and 5.1 mm for 6, 12, and 20 MeV, respectively.

#### A.2 Comparing R50,D measured using the MatriXX 2D ion chamber array vs. calculated using eMC

**Figure 2:**
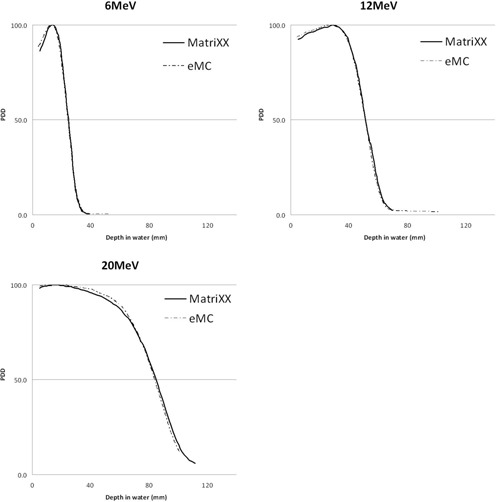
Percentage depth‐dose curves obtained using the MatriXX in solid water and the eMC‐calculated PDD curves in solid water at 6, 12, and 20 MeV. The energy‐dependent inherent buildup shifts calculated are applied to the PDD curves obtained with the MatriXX.

The depth of $R_{50,D}$ was measured from the curves shown in Fig. 2. It was found that the position of $R_{50,D}$ measured using the MatriXX agreed to within 1 mm against eMC values for all energies (−0.5 mm, 0.6 mm, and −0.9 mm for 6, 12, and 20 MeV, respectively).

When compared to the commissioning values measured using an NACP chamber in water, it is found that the position of $R_{50,D}$ measured using the MatriXX (with the inherent buildup correction applied) agrees to within 0.5 mm (−0.3 mm,−0.4 mm, and −0.4 mm for 6, 12, and 20 MeV, respectively).

### B. Dose plane verification using the MatriXX 2D ion chamber array

**Figure 3:**
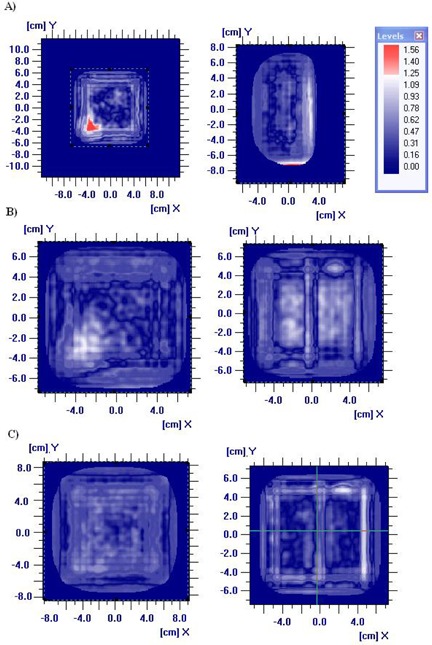
Gamma map comparing the dose distributions at 6 (a), 12 (b), and 20 (c) MeV in a homogeneous solid water phantom (left) and a heterogeneous solid water phantom with a lung insert (right).

Using the homogeneous and heterogeneous phantoms described above, dose planes were measured using the MatriXX and compared with the equivalent dose planes calculated using the Eclipse eMC algorithm. Gamma maps can be seen in Fig. 3 for 6 MeV (3(a)), 12 MeV (3(b)), and 20 MeV (3(c)). The gamma analysis results (3%/3 mm) are shown in Table 3.

**Table 3:** Gamma analysis results at 3%/3 mm and 2%/2 mm comparing the dose planes measured using the with the dose planes calculated by the eMC algorithm in Eclipse.

	3%/3 mm		2%/2 mm	
*Energy (MeV)*	*Homogeneous Solid Water Phantom*	*Heterogeneous Solid Water Phantom (with lung insert)*	*Homogeneous Solid Water Phantom*	*Heterogeneous Solid Water Phantom (with lung insert)*
6	97.0%	98.9%	90.1%	92.4%
12	99.3%	98.4%	95.7%	95.1%
20	100%	99.1%	99.3%	92.4%

It can be seen from Table 3 that the gamma analysis results at 3%/3 mm show very good agreement between the dose distributions measured using the MatriXX and those calculated using eMC, for both heterogeneous and homogeneous phantoms. However at 2%/2 mm, which is the level at which the Gafchromic film shows good agreement with the eMC algorithm, due to the limitation of the spatial resolution of the MatriXX it is evident that there isn't as good agreement. These results demonstrate that the MatriXX does not perform as well as the Gafchromic film in verifying dose distributions calculated by the eMC algorithm.

The gamma map shown in Fig. 3(a) for the 6 MeV solid water map, shows a large area where the gamma analysis >1. It was found upon inspection of the solid water used, that there was a defect in the solid water slab which meant that there was a large disagreement in the area of the defect. This isn't seen in all of the dose maps as the solid water phantom was made up of smaller slabs and only the very superficial slab had the defect.

It can also be seen from Table 3 that the percentage of points passing the gamma analysis is poorer for lower energies. This could be due to the depth at which the measurement was taken and a lack of scatter due to the shallow depth of measurement.

## IV. CONCLUSION

Once suitably calibrated with the inherent buildup characterized, the MatriXX Im'*RT* 2D ion chamber array has been shown to be suitable for performing the verification measurements required to assess the functionality of the Eclipse eMC algorithm. However, it does not perform as well as Gafchromic film when verifying dose distributions in homogeneous and heterogeneous phantoms in standard setups. Limitation in the spatial resolution of the MatriXX may have caused poor pass rate for the criterion of 2%/2 mm.

For this reason it is suggested that Gafchromic film remains the gold standard for TPS commissioning verification measurements, but in centers which may not have a Gafchromic service available to them, the results show that the MatriXX can provide a suitable alternative to perform the measurements required to verify the eMC algorithm.

The ease of setup and use makes the MatriXX suitable not only for initial verification of the eMC algorithm but also for periodic QA checks, as well as verification measurements following a TPS upgrade.

## ACKNOWLEDGMENTS

Mark D. Wanklyn would like to thank all of the staff at Queen's Hospital, Romford, for all of their help and support throughout his time there, without which this publication would not have been possible.

## COPYRIGHT

This work is licensed under a Creative Commons Attribution 3.0 Unported License.
